# Dietary licorice flavonoids powder improves serum antioxidant capacity and immune organ inflammatory responses in weaned piglets

**DOI:** 10.3389/fvets.2022.942253

**Published:** 2022-07-26

**Authors:** Shenggang Yin, Ting You, Jiayong Tang, Longqiong Wang, Gang Jia, Guangmang Liu, Gang Tian, Xiaoling Chen, Jingyi Cai, Bo Kang, Hua Zhao

**Affiliations:** ^1^Key Laboratory for Animal Disease-Resistance Nutrition, Ministry of Education, Ministry of Agriculture and Rural Affairs, Animal Nutrition Institute, Sichuan Agricultural University, Chengdu, China; ^2^College of Animal Science and Technology, Sichuan Agricultural University, Chengdu, China

**Keywords:** licorice flavonoids powder (LFP), antioxidant ability, immunity, biochemical parameters, piglets

## Abstract

Weaning often induces oxidative stress and inflammatory response in piglets. This study investigated the effects of dietary licorice flavonoids powder (LFP) supplementation on antioxidant capacity and immunity in weaned piglets. Notably, 96 Landrace × Yorkshire × Duroc (DLY) weaned piglets were randomly allocated to four treatments with 6 replicates (4 animals per replicate) and fed with diet supplementation with 0, 50, 150, and 250 mg/kg LFP, respectively. The trial lasted for 5 weeks. The results showed that dietary LFP supplementation effectively increased the liver index (*P* < 0.05). In addition, dietary LFP supplementation reduced serum aspartate aminotransferase activity (*P* < 0.01). Piglets fed with 50 mg/kg LFP decreased total cholesterol and HDL-C content in serum (*P* < 0.05) and increased serum alkaline phosphatase activity (*P* < 0.01). Similarly, supplementation with 150 mg/kg LFP elevated the activity of total antioxidant capability (T-AOC) in serum (*P* < 0.01) and dietary with 150 and 250 mg/kg LFP increased T-AOC activity in spleen (*P* < 0.01). Moreover, dietary with 150 mg/kg LFP addition enhanced (*P* < 0.05) the serum IgG content of piglets. Additionally, compared with the control group, dietary 250 mg/kg LFP supplementation upregulated (*P* < 0.05) the mRNA abundance of *Interleukin* (*IL)-1*β and *monocyte chemoattractant protein 1 (MCP-1)* in the spleen. Meanwhile, dietary 150 and 250 mg/kg LFP supplementation downregulated (*P* < 0.05) mRNA abundance of *IL-10*, and *MCP-1* and 250 mg/kg LFP upregulated (*P* < 0.05) the expression of *intercellular adhesion molecule 1 (ICAM-1), IL-1*β, *IL-6*, and *tumor necrosis factor* α *(TNF-*α*)* in the thymus. In conclusion, LFP supplementation improved the immune function of piglets by regulating the activity of serum biochemical enzymes, improving the antioxidant capacity, and alleviating inflammation of immune organs. This study indicated that LFP is potential alternative protection against early weaned stress in piglets.

## Introduction

Piglets encounter nutritional, physiological, and environmental stressors after weaning that often are accompanied by reduced pig health and increased immune system dysfunctions (1). Significant changes in the immunology of piglets include reduced production of antibodies and depressed function of cellular immunity (2). The immune function is one of the defense mechanisms against infection in the animal body and is closely related to inflammation, which is caused by the activation of immune cells in local tissues. The inflammatory response can be regulated by the expression of cytokines, such as interleukin (IL)-1β, IL-6, and tumor necrosis factor-α (TNF-α) (3).

Weaning stress disrupted free-radical metabolism and the antioxidative system and caused serious oxidative stress (4). Under normal physiological conditions, the generation and scavenging of free radicals are in a balanced state, and the disturbance of the balance will lead to oxidative stress (5). Antioxidant enzymes, including glutathione peroxidase (GSH-Px) and total superoxide dismutase (T-SOD), neutralize toxic oxygen products to normalize the balance of the homeostasis of the body (5).

In the past decades, antibiotics, as medicine and growth promoters, have been commonly applied in animal production. Due to increased antibiotic residues and bacterial resistance as well as the ban on using antibiotics in the feed industry in China and Europe, a growing interest in natural substances as alternatives to antibiotics (6). Licorice, with many bioactive compounds such as flavonoids and glycyrrhizin, has been frequently used as herbal medicine to suppress peroxidation and inflammation. This study has shown that chalcones A and B inhibit lipid peroxidation of rat liver microsomes and dose-dependently inhibit lipopolysaccharide-induced macrophage reactive oxygen production (7). Simultaneously, licorice chalcone inhibits mitochondrial lipid peroxidation (8) and has a strong inhibitory effect on the production of superoxide anions and scavenging activity on free radicals (9). Previous research indicated that licorice and its extract reduced the release of nitric synthase (*INOS*) and cyclooxygenase-2 (*COX-2*) and the mRNA expression of pro-inflammatory cytokines *TNF-*α, *IL-6*, and *IL-1*β in lipopolysaccharide (LPS)-treated macrophages and mouse model (10, 11). Therefore, licorice flavonoids powder (LFP) was a substance derived from licorice after the extraction process exerting a potential role in enhancing the antioxidant capacity and immune function of animals.

Our study indicated that dietary LFP supplementation has a trend to improve the performance of weaning piglets (12). The subsequent question was, therefore, to find whether those improvements be partly attributed to the enhancement of the antioxidant capacity and immune function of weaned piglets fed on feeds free of antibiotics by the dietary LFP inclusion. Therefore, this study aimed to assess the beneficial effects of dietary LFP supplementation on weaning piglets, biochemical parameters, immunoglobulin content, antioxidant capacity, and mRNA expression of inflammation-related genes in serum or tissues.

## Materials and methods

### Animals, diet, and experimental design

The experiment followed the actual law of animal protection and was approved by the Animal Care and Use Committee of the Sichuan Agricultural University (Ethics Approval Code: SCAUAC201811-1). This study used the same growth experiment as our previous research (12). A total of 96 piglets (Duroc × Landrace × Yorkshire, DLY) were weaned at 28 days with an initial average body weight of 8.05 ± 0.23 kg and were randomized to 4 dietary treatments with 6 replicates (*n* = 4 animals per replicate). Weaned piglets fed on a basal diet (BD) or BD supplemented with 50, 150, or 250 mg/kg LFP. The BD (Supplementary Table 1) was matched the formulation reported previously (13) and provided in powder form. LFP was added to the basal diet by step-by-step mixing. LFP was derived from the residue after water extraction from *Glycyrrhiza inflata Bat., Glycyrrhiza uralensis Fisch*., and *Glycyrrhiza glabra L*. LFP, which contained flavonoid (60%) and glycyrrhizin (10%), was provided by Kaimeijia Biotechnology Company (Xinjiang, China). Diets were formulated according to the criteria recommended by NRC2012 (14). The pigs received feed and water on an *ad libitum* basis throughout the trial. The trial lasted for 35 days. The room temperature was maintained at 28 ± 1 °C throughout the trial.

### Sample collection and preparation

Dietary LFP inclusion tended to increase the average daily gain (ADG) and reduce the feed intake/body gain (F/G) of piglets (12). At the end of the experiment (35 days), after 8 h of fasting, six pigs per group with average body weight were selected, and body weight was recorded. The blood samples were collected from the vena cava anterior and then euthanized by a lethal injection of pentobarbital sodium. Liver, spleen, and thymus tissues were weighed and divided into aliquots using surgical scissors. The organ index was calculated according to the organ weight and the body weight of piglets. The tissue samples were snap-frozen in liquid nitrogen and stored at −80°C until use. Serum samples were prepared by centrifugation of the whole blood at 3,000 × *g* for 15 min at 4 °C with anticoagulant-free tubes and stored at −20°C.

### Serum biochemical analyses

The activities of serum aspartate aminotransferase (AST), alanine aminotransferase (ALT), serum alkaline phosphatase (ALP), gamma-glutamyl transpeptidase (GGT), lactate dehydrogenase (LDH), and the concentrations of total protein (TP), albumin (ALB), glucose (GLU), total cholesterol (TC), triglyceride (TG), high-density lipoprotein cholesterol (HDL-C), low-density lipoprotein cholesterol (LDL-C), and blood urea nitrogen (BUN) were determined using an automatic biochemistry radiometer (3100, HITACHI, Japan).

### Antioxidant capability analyses

The concentration of malondialdehyde (MDA) and the activities of total antioxidant capability (T-AOC), T-SOD, and GSH-Px in serum, liver, and spleen were measured using commercial assay kits (No. A015-1-2, A003-1-2, A001-1-2, and 005-1-2, Nanjing Jiancheng, China) according to the manufacturer's instruction. In brief, ~0.1 g of the liver or spleen sample was homogenized at a ratio of 1:9 (w/v) with ice-cold phosphate-buffered saline. Homogenate was centrifuged at 3,000 × *g* for 10 min at 4 °C to obtain supernatant and then immediately applied to the analysis. The concentration of protein was determined using the bicinchoninic acid (BCA) protein assay kit (No. D1001-A, MeiMian, China). The optical density (OD) values were monitored using a Gemini EM Microplate Reader (SpectraMax® Gemini XPS, Molecular Devices, San Jose, CA, USA).

### Serum immunoglobulin analyses

Serum immunoglobulins, i.e., IgA, IgG, and IgM, were measured using ELISA assay kits (No. MM-090502, M M-040302, and MM-040202, MeiMian, China) according to the manufacturer's instructions. In brief, the standard sample or serum diluted sample was added to the enzyme plate and incubated at 37 °C for 30 min. The HRP-conjugate reagent was added to the enzyme plate and incubated at 37 °C for 30 min after washing 5 times. Then, chromogen solutions A and B were added to the enzyme plate and incubated at 37 °C for 30 min after washing 5 times. Subsequently, the absorbance was read at 450 nm within 15 min after adding the stop solution.

### Real-time QPCR analyses

The total RNA of liver, spleen, and thymus samples (~0.1 g) was extracted using the Trizol (No. 15596018, Invitrogen, USA) method, and the concentration and purity of the total RNA were measured at 260 and 280 nm using a spectrophotometer (NanoDrop 2000, Thermo Fisher Scientific, Waltham, MA, USA). The cDNA was synthesized using the PrimeScript RT reagent kit (No. RR047A, TaKaRa, China). Real-time qPCR was performed on QuantStudio 6 Flex system (Applied Biosystems, USA) using the SYBR Premix Ex Taq™ II kit (No. RR820A, TaKaRa, China). The primers (Supplementary Table 2) for 9 inflammation-related genes and 2 reference genes (β-*ACTIN* and *GAPDH*) were designed using Primer Express 3.0 (Applied Biosystems, USA). The relative mRNA abundance was quantified as previously described using the 2^−ΔΔCt^ method (15).

### Statistical analysis

The experiment was a completely random design and applied the one-way structure treatment design. The linear-quadratic regression equation was used to estimate the effects of dietary LFP levels using the MIXED procedure of SAS9.4 (16). Multiple treatment comparisons followed the Tukey's test subjected to the LSMEAN statement of SAS 9.4 (16). The normality and homogeneity of variance were evaluated using the Shapiro-Wilk W test and Levene's using the UNIVARIATE and HOVTEST statements. All data were presented as the mean and the standard error of means (SEM), and statistical differences in statistics were considered as *P* ≤ 0.05 and tendencies at 0.05 < *P* ≤ 0.10.

## Results

### Effect of LFP supplementation on organ index of weaned piglets

As shown in Table 1, the liver index elevated linearly (*P* < 0.05) and quadratically (*P* = 0.064) with increasing LFP supplementation in the diet. The piglets supplemented with LFP 50, 150, and 250 mg/kg showed increased liver index compared with those receiving LFP 0 mg/kg. But only a significantly higher liver index was observed in the piglets receiving LFP 250 mg/kg compared with the control group (*P* < 0.05). There was no significant difference in the index of the spleen, thymus, and kidney of piglets among all the treatments (*P* > 0.10).

**Table 1:** Organ index^*^ of piglets fed with diets containing different levels of LFP.

**Items**	**Dietary supplemented LFP level, mg/kg**				**SEM**	***P*** **-value**		
	**0**	**50**	**150**	**250**		**ANOVA**	**Linear**	**Quadratic**
Liver	24.92^b^	27.19^ab^	26.21^ab^	29.96^a^	0.62	0.017	0.033	0.064
Spleen	2.15	2.00	2.10	2.22	0.10	0.776	0.309	0.906
Thymus	0.97	0.72	0.83	0.92	0.04	0.105	0.048	0.709
Kidney	5.47	5.20	4.97	5.50	0.14	0.514	0.825	0.731

*Organ index (g/kg), organ weight (g)/body weight (kg). SEM, total standard error of the means (n = 6). LFP, licorice flavonoids powder. ^a, b^Mean values within a row with different superscript letters were significantly different among diets with 0, 50, 150, and 250 mg/kg of LFP (one-way ANOVA, P < 0.05, Tukey's post-hoc test).

### Effect of LFP supplementation on serum biochemical of weaned piglets

As presented in Table 2, dietary LFP supplementation had a linear and quadratic decreased effect on the AST activity in the serum (*P* < 0.01). The piglets receiving LFP exhibited significantly lower AST activity in the serum compared with the control group (*P* < 0.01). Then, we investigated the indices of lipid metabolism of serum. LFP manipulation linearly increased ALP activity and decreased TC and HDL-C content (*P* < 0.01). Furthermore, dietary with 50 mg/kg LFP inclusion significantly elevated ALP activity compared with the control group (*P* < 0.01). In addition, the piglets receiving LFP 50 mg/kg showed lower TC (*P* < 0.05) and HDL-C content (*P* < 0.05) to the control group. However, no differences have been seen in the activities of ALT, GGT, and LDH and the contents of TP, ALB, GLOB, GLU, TG, LDL-C, and BUN (*P* > 0.10).

**Table 2:** Serum biochemical parameters of piglets fed with diets containing different levels of LFP.

**Items**	**Dietary supplemented LFP level, mg/kg**				**SEM**	***P*** **-value**		
	**0**	**50**	**150**	**250**		**ANOVA**	**Linear**	**Quadratic**
ALT (U/L)	29.00	33.80	32.00	29.00	1.42	0.578	0.380	0.764
AST (U/L)	57.50^a^	38.20^b^	38.83^b^	41.50^b^	2.25	0.001	0.002	0.006
TP (g/L)	55.83	54.77	54.93	55.25	0.52	0.921	0.583	0.860
ALB (g/L)	26.50	25.72	26.52	25.30	0.38	0.799	0.452	0.835
GLOB (g/L)	30.37	29.67	28.42	28.37	0.81	0.874	0.779	0.348
ALP (U/L)	115.20^b^	184.40^a^	154.80^ab^	156.00^ab^	7.98	0.009	0.002	0.654
GGT (U/L)	33.67	33.80	34.67	30.00	1.82	0.823	0.810	0.719
GLU (mmol/L)	4.56	4.26	4.65	4.08	0.14	0.531	0.260	0.880
TC (mmol/L)	2.29^a^	1.97^b^	2.13^ab^	2.06^ab^	0.04	0.042	0.007	0.642
TG (mmol/L)	0.40	0.38	0.40	0.40	0.02	0.980	0.708	0.837
HDL-C (mmol/L)	0.68^a^	0.56^b^	0.65^ab^	0.62^ab^	0.02	0.017	0.002	0.691
LDL-C (mmol/L)	0.85	0.91	0.93	0.90	0.02	0.611	0.490	0.402
LDH (U/L)	313.36	300.66	271.52	304.53	11.78	0.612	0.963	0.451
BUN (mmol/L)	1.30	1.24	1.18	1.36	0.08	0.727	0.961	0.806

*SEM, total standard error of the mean (n = 6). LFP, licorice flavonoids powder; ALT, alanine aminotransferase; AST, aspartate aminotransferase; TP, total protein; ALB, albumin; GLOB, globulin, GLOB = TP – ALB; ALP, alkaline phosphatase; ALP, alkaline phosphatase; GGT, gamma-glutamyl transpeptidase; GLU, glucose; TC, total cholesterol; TG, triglyceride; HDL-C, high-density lipoprotein cholesterol; LDL-C, low-density lipoprotein cholesterol; LDH, lactate dehydrogenase; BUN, blood urea nitrogen. ^a, b^Mean values within a row with different superscript letters were significantly different among diets with 0, 50, 150, and 250 mg/kg of LFP (one-way ANOVA, P < 0.05, Tukey's post-hoc test)*.

### Effect of LFP on antioxidant capacity of serum, liver, and spleen in weaned piglets

As shown in Table 3, we investigated 3 well-known antioxidant activities and MDA content. Dietary LFP supplementation quadratically increased T-AOC in the serum (*P* < 0.01) and spleen (*P* < 0.01) and elevated GSH-Px in the liver (*P* = 0.094) and spleen (*P* < 0.05). Compared with pigs in the control group, dietary with 150 mg/kg LFP supplementation significantly elevated the activity of T-AOC in serum (*P* < 0.01). Moreover, piglets with 150 and 250 mg/kg LFP supplementation showed remarkably higher T-AOC activity in the spleen compared with 0 and 50 mg/kg LFP treatment groups (*P* < 0.01). Furthermore, there existed a trend to elevate the activity of GSH-Px in the liver (*P* = 0.089) and spleen (*P* = 0.080) when piglets were supplemented with LFP. However, T-SOD activity and MDA content among the four treatments in serum, liver, and spleen were not different (*P* > 0.10).

**Table 3:** Antioxidant capacity of serum, liver, and spleen of piglets fed with diets containing different levels of LFP.

**Items**	**Dietary supplemented LFP level, mg/kg**				**SEM**	***P*** **-value**		
	**0**	**50**	**150**	**250**		**ANOVA**	**Linear**	**Quadratic**
**Serum**								
T-AOC (U/mL)	1.10^b^	1.21^b^	1.83^a^	1.57^ab^	0.09	0.007	0.939	0.001
MDA (nmol/mL)	2.98	2.38	2.41	2.33	0.10	0.106	0.042	0.122
T-SOD (U/mL)	119.46	135.36	124.67	124.38	1.89	0.105	0.027	0.637
GSH-P_X_ (U/mL)	764.74	682.13	808.10	742.52	17.70	0.284	0.102	0.316
**Liver**								
T-AOC (U/mL)	0.69	0.77	0.80	0.81	0.02	0.384	0.321	0.170
MDA (nmol/mL)	0.83	0.77	0.63	0.78	0.04	0.356	0.938	0.292
T-SOD (U/mL)	470.81	478.43	470.55	497.46	9.30	0.724	0.577	0.637
GSH-P_X_ (U/mL)	585.26	603.47	604.07	676.23	14.21	0.089	0.279	0.094
**Spleen**								
T-AOC (U/mL)	0.76^b^	0.81^b^	1.44^a^	1.29^a^	0.84	0.001	0.997	0.000
MDA (nmol/mL)	1.08	1.07	0.99	0.79	0.06	0.400	0.871	0.224
T-SOD (U/mL)	7.57	7.86	7.62	7.09	0.20	0.376	0.985	0.670
GSH-P_X_ (U/mL)	542.22	544.27	599.62	559.00	9.19	0.080	0.665	0.046

*SEM, total standard error of the mean (n = 6). LFP, licorice flavonoids powder; T-AOC, total antioxidant capability; MDA, malondialdehyde; T-SOD, total superoxide dismutase; GSH-Px, glutathione peroxidase. ^a, b^Mean values within a row with different superscript letters were significantly different among diets with 0, 50, 150, and 250 mg/kg of LFP (one-way ANOVA, P < 0.05, Tukey's post-hoc test)*.

### Effect of LFP supplementation on serum immunoglobulin levels

We investigated serum immunoglobulin levels in Table 4. Considering the immunoglobulin in the serum, quadratic responses were observed in serum IgG to dietary LFP supplementation (*P* < 0.01). Compared with the control group, the supplementation of 150 mg/kg LFP increased (*P* < 0.05) the serum IgG content of piglets. LFP administration did not show any significant effect on serum IgA and IgM (*P* > 0.05).

**Table 4:** Serum immunoglobulin levels of piglets fed with diets containing different levels of LFP.

**Items**	**Dietary supplemented LFP level, mg/kg**				**SEM**	***P*** **-value**		
	**0**	**50**	**150**	**250**		**ANOVA**	**Linear**	**Quadratic**
IgA (μg/mL)	39.95	38.41	38.34	41.26	1.46	0.603	0.956	0.334
IgG (μg/mL)	423.17^b^	446.54^ab^	514.70^a^	485.60^ab^	12.59	0.043	0.660	0.009
IgM (μg/mL)	35.55	40.22	41.23	38.19	1.65	0.657	0.192	0.656

*SEM, total standard error of the mean (n = 6). LFP, licorice flavonoids powder; IgA, immunoglobulin A; IgG, immunoglobulin G; IgM, immunoglobulin M. ^a, b^Mean values within a row with different superscript letters were significantly different among diets with 0, 50, 150, and 250 mg/kg of LFP (one-way ANOVA, P < 0.05, Tukey's post-hoc test)*.

### Effect of LFP supplementation on MRNA abundance of inflammation-related genes

**Figure 1:**
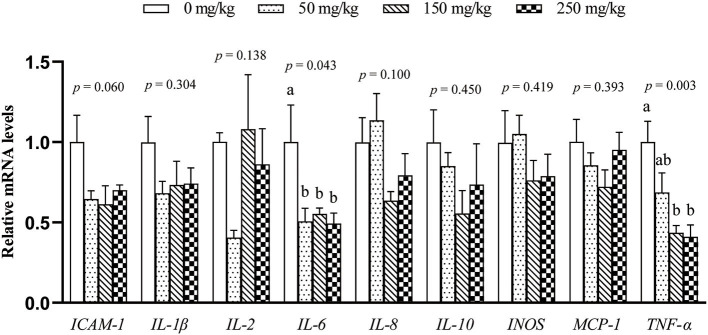
Effect of LFP on mRNA abundance of inflammation-related genes in the liver of weaned piglets. Data are presented as means ± SEM (*n* = 6). ^a, b^Mean values with unlike letters were significantly different (*P* < 0.05). LFP, licorice flavonoids powder; *ICAM-1*, intercellular adhesion molecule 1; *IL-1*β, interleukin 1β; *IL-2*, interleukin 2; *IL-6*, interleukin 6; *IL-8*, interleukin 8; *IL-10*, interleukin 10; *INOS*, inducible nitric oxide synthase; *MCP-1*, monocyte chemoattractant protein 1; *TNF-*α, tumor necrosis factor α.

**Figure 2:**
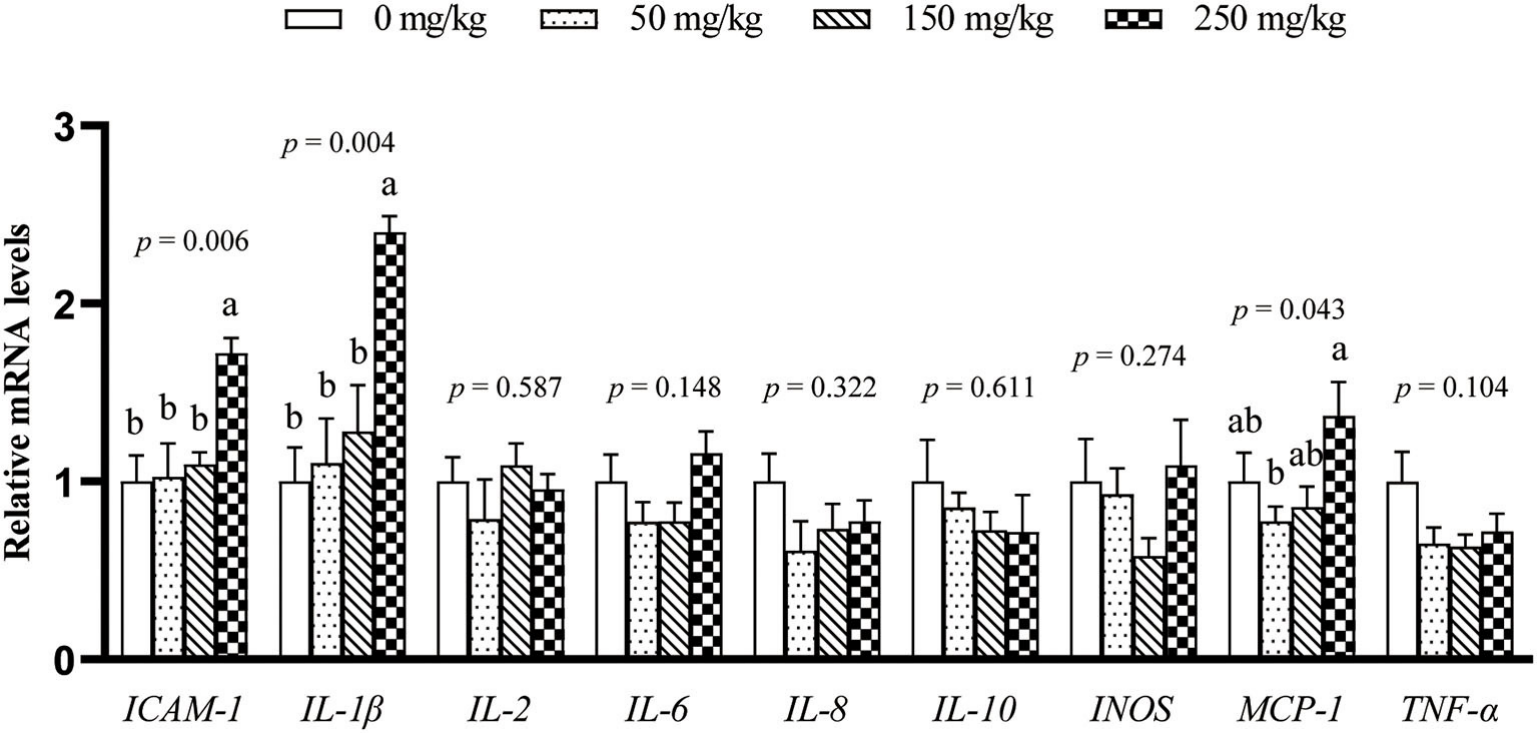
Effect of LFP on mRNA abundance of inflammation-related genes in the spleen of weaned piglets. Data are presented as means ± SEM (*n* = 6). ^a, b^Mean values with unlike letters were significantly different (*P* < 0.05). LFP, licorice flavonoids powder; *ICAM-1*, intercellular adhesion molecule 1; *IL-1*β, interleukin 1β; *IL-2*, interleukin 2; *IL-6*, interleukin 6; *IL-8*, interleukin 8; *IL-10*, interleukin 10; *INOS*, inducible nitric oxide synthase; *MCP-1*, monocyte chemoattractant protein 1; *TNF-*α, tumor necrosis factor α.

**Figure 3:**
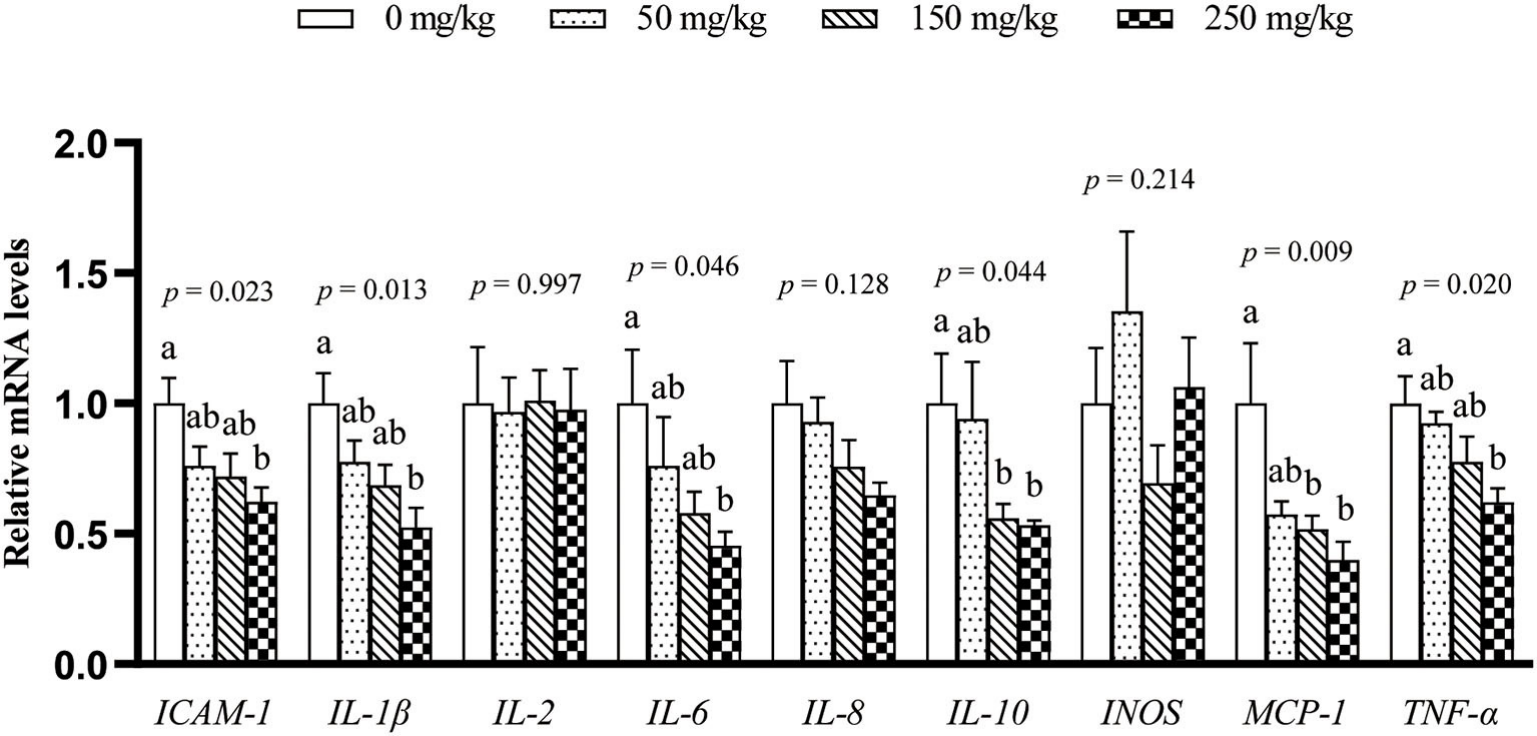
Effect of LFP on mRNA abundance of inflammation-related genes in the thymus of weaned piglets. Data are presented as means ± SEM (*n* = 6). ^a, b^Mean values with unlike letters were significantly different (*P* < 0.05). LFP, licorice flavonoids powder; *ICAM-1*, intercellular adhesion molecule 1; *IL-1*β, interleukin 1β; *IL-2*, interleukin 2; *IL-6*, interleukin 6; *IL-8*, interleukin 8; *IL-10*, interleukin 10; *INOS*, inducible nitric oxide synthase; *MCP-1*, monocyte chemoattractant protein 1; *TNF-*α, tumor necrosis factor α.

The mRNA expression of 9 inflammation-related genes (*ICAM-1, IL-1*β, *IL-2, IL-6, IL-8, IL-10, MCP-1, TNF-*α, and *INOS*) in the liver was determined (Figure 1). Compared with the control group, dietary LFP inclusion downregulated the mRNA levels of *IL-6, TNF-*α (*P* < 0.05), and *ICAM-1* (*P* = 0.06) in the liver. Moreover, dietary LFP supplementation also affected the expression of inflammation-related genes in the spleen of piglets (Figure 2). Compared with the control group, dietary 250 mg/kg LFP supplementation upregulated (*P* < 0.05) the mRNA profiles of *IL-1*β and *MCP-1* in the spleen. Furthermore, dietary LFP supplementation exhibited a great impact on the expression of inflammation-related genes in the thymus (Figure 3). The mRNA expression of *ICAM-1, IL-1*β, *IL-6*, and *TNF-*α was lower in the 250 mg/kg LFP supplementation group than in the control group (*P* < 0.05). Dietary 150 and 250 mg/kg LFP supplementation downregulated the mRNA expression of *IL-10* and *MCP-1* compared with the control group. In general, LFP supplementation suppresses the expression of inflammation-related genes in the liver and thymus, thus enhancing the immunity function of weaned piglets.

### Estimation of the dietary LFP recommended level

According to different effect indexes, the recommended levels of LFP through quadratic regression analysis and the establishment of the univariate quadratic equation are shown in Table 5. Under the condition of this experiment, based on serum AST activity, serum and spleen T-AOC activity, spleen GSH-Px, and serum IgG content as the effect index, the suitable supplementation of LFP in the basic diet of piglets were 158, 205, 175, 153, and 170 mg/kg LFP, respectively.

**Table 5:** The optimal dietary LFP supplementation based on different indices for piglets.

**Items**	Regression equation*	**R** ^2^	***P*** **-value**	**Optimal dietary LFP levels (mg/kg diet)**
AST activity in serum (U/L)	y = 0.0008x^2^-0.2528x + 54.469	0.770	0.006	158
T-AOC activity in serum (U/mL)	y = −0.00002x^2^ + 0.0082x + 1.0195	0.860	0.001	205
T-AOC activity in spleen (U/mL)	y = −0.00002x^2^ + 0.007x + 0.6721	0.862	0.000	175
GSH-Px activity in spleen (U/mL)	y = −0.0024x^2^ + 0.733x + 532.31	0.713	0.046	153
IgG of content in serum (μg/mL)	y = −0.0031x^2^ + 1.0567x + 415.58	0.928	0.009	170

*y is the dependent variable and x are the dietary LFP supplemental levels (mg/kg); LFP, licorice flavonoids powder.

*SEM, total standard error of the mean (n = 6). Mean values within a row with different superscript letters were significantly different among diets with 0, 50, 150, and 250 mg/kg of LFP (one-way ANOVA, P < 0.05, Tukey's post-hoc test)*.

## Discussion

Weaning stress decreases growth performance and damages the immune function of piglets (1). Supplementation of plant extracts improves growth performance, alleviates oxidant stress, and improves immune function, thereby reducing the negative effects of weaning stress on piglets (17). Licorice or its extract exhibits antioxidant function (8, 9) and immunomodulatory function on RAW264.7 cells and mice (11, 18, 19).

Serum biochemical parameters reflect the metabolism of substances in the animal body. We investigated dietary LFP supplementation on serum biochemical parameters of piglets. Previous studies have shown that licorice extract mainly affects the index of the liver and intestine of weaned piglets (18, 20). In this study, LFP supplementation reduced the AST levels in the serum of piglets (Table 2). Transaminase plays an important role in the process of amino acid metabolism, and AST acts as an indicator intracellular enzyme relative to liver function and is used as the index of liver damage (21). A reduced serum AST level indicated improvement of the liver function of weaned piglets. Studies have revealed that licorice extract or its active ingredient glycyrrhizic acid has a hepatoprotective function, and licorice extract inhibited the increase of serum AST and ALT in a liver injury rat model induced by carbon tetrachloride (22). Glycyrrhetinic acid, a decomposition product of glycyrrhizic, protects liver cells from tert-butyl hydroperoxide-induced oxidant damage by inhibiting the reduction of intracellular glutathione and reactive oxygen species and depolarizing the mitochondrial membrane (23). Therefore, it is not strange that LFP increased the liver index of weaned piglets (Table 1), indicating a protective effect of LFP on liver function. The activity of serum ALP in serum reflects the metabolic efficiency of lipids. ALP is an isozyme with genetic markers in serum. Animal performances are affected by ALP activity, and daily body weight gain is positively correlated with ALP activity (24). It is more than just that serum cholesterol reflects the absorption and metabolism of lipids by the animal body. Notably, 50 mg/kg LFP reduced TC and HDL-C in serum (Table 2), and similar results are found that supplementation of licorice extract through drinking water reduces the serum glucose, LDL-C, and total cholesterol in broilers (25). This decrease in serum TC and HDL-C may be related to the inhibition of lipid peroxidation by licorice, it inhibits the formation of lipid peroxides, and in the subsequent liver, the clearance process increases the rate of conversion of cholesterol into bile acids, thereby reducing cholesterol and increasing liver bile acid content (25). These results suggested that dietary LFP supplementation can improve the health status of piglets by regulating the activity of serum biochemical enzymes.

Weaning stress results in oxidant stress, which may induce a variety of diseases. We explored the effect of dietary LFP on the antioxidant capacities in serum, liver, and spleen. Dietary LFP supplementation moderately increased the activities of GSH-Px, SOD, and T-AOC and decreased the MDA level in those tissues (Table 3). The antioxidant activity of LFP may be attributed to its chemical components, including glycyrrhizin flavonoids, glycyrrhizic, and polysaccharides (26, 27), which have multiple phenolic hydroxyl groups and benzene ring structures. Chalcone C attenuates the inflammation induced by lipopolysaccharide and interferon by reducing the expression of inducible NO and regulating the activity of antioxidant enzymes such as GSH-Px (28). Glycyrrhizin inhibits mitochondrial lipid peroxidation and scavenges free radicals (8), and chalcone has a strong inhibitory effect on the production of superoxide anions and has a strong scavenging activity on free radicals. The mechanism of antioxidant capacity of flavonoid compounds includes inhibiting the generation of reactive oxygen species (ROS) by affecting their conformation and inhibiting enzyme activity involved in their production, scavenging of ROS, or upregulation or protection of antioxidant defense (29, 30). Furthermore, flavonoids obviously inhibit the formation of MDA and have a strong scavenging effect on hydroxyl radical and superoxide anion radical (26). In this study, LFP contains 60% flavonoids; therefore, supplementation of dietary LFP enhanced the antioxidant capacity of weaned piglets.

Interleukin-6, IL-1β, and TNF-α are important mediators of the inflammatory response, which were commonly used as markers for systemic pro-inflammatory cytokine activation and important inducers of acute-phase proteins (31). IL-2 is known to be associated with cellular immunity and plays an important role in animal immune function (32). IL-8 plays a key role in the recruitment and activation of neutrophils during inflammation (33). The immune response can be regulated by the expression of INOS, ICAM-1, and MCP-1 (34, 35), and ICAM-1 is an adhesion molecule that promotes the firm adhesion of leukocytes to endothelial cells (36). ICAM-1 and MCP-1 have a low basal expression in epithelial cells but are upregulated in response to a variety of inflammatory mediators (37). The downregulation of those inflammation-related genes in the liver, spleen, and thymus indicated enhancement of immunity function of the weaned piglets by LFP. Licorice extract reduces the secretion and mRNA levels of *TNF-*α, *IL-6*, and *IL-1*β in macrophages and inhibits skin swelling and the expression of *INOS* and *COX-2* in the mouse inflammation model (10). *Glycyrrhiza uralensis* reduces the release of NO and PGE2 in LPS-treated macrophages and downregulates mRNA levels of *IL-6, TNF-*α, *IL-1*β, and *COX-2* (18). Chalcone A, the active ingredient of flavonoids in LFP, inhibits the release of cytokines in mice (38). IL-10 is an anti-inflammatory cytokine associated with humoral immunity (39). The downregulation of the expression of *IL-10* in the thymus by LFP (Figure 3) may indicate that piglets were in a healthy condition and did not need to express more IL-10 to maintain immune homeostasis (Figure 3).

We further investigated the serum immunoglobulin levels of piglets. IgG accounts for ~80% of the total serum immunoglobulin and plays a role in resisting the invasion of viral, bacterial, and fungal infections (40). In this study, LFP increased the serum IgG (Table 4). The increase of serum IgG indicated enhancement of the serum immunity of weaned piglets by dietary LFP. The active ingredients in LFP, such as glycyrrhizin flavonoids and glycyrrhizic acid, increase the phagocytic function of phagocytic cells, regulate the number and function of lymphocytes (41), thus preventing some inflammatory mediators by regulating immune-related signaling pathways or enzyme activities produced to activate macrophages, and indirectly inhibit platelet aggregation (41), inflammatory cytokine secretion (42, 43), and neutrophil adhesion (36), thus regulating the inflammatory response and improving the immune function of piglets.

In conclusion, dietary supplementation with LFP regulates serum biochemistry enzyme activity, promotes liver metabolic function, improves the antioxidant capacity, inhibits the expression of inflammation-related genes, and enhances the serum IgG content, thus promoting the health status of weaned piglets. In this study, the optimal LFP levels for improving the health status of piglets were determined to be 150 mg/kg based on the serum and immune organ indices.

In this study, our result indicates that dietary LFP supplementation improves the immunity of weaned piglets; however, more studies need to be conducted to further explore the detailed mechanism relating to immunity regulation roles of the substance obtained from licorice, a traditional Chinese herbal medicine, thus providing new insight on the application of those plant extracts in pig product in the future, especially in weaning piglets faced with different stress challenges.

## Data availability statement

The original contributions presented in the study are included in the article/Supplementary material, further inquiries can be directed to the corresponding author.

## Ethics statement

The animal study was reviewed and approved by the animal experiment is followed the actual law of animal protection and was approved by the Animal Care and Use Committee of the Sichuan Agricultural University (Ethics Approval Code: SCAUAC201811-1).

## Author contributions

HZ, TY, and SY designed the research and wrote the manuscript. TY and SY conducted the experiments. JT, LW, GJ, GL, XC, GT, JC, and BK collected sample and analyzed the data. HZ had primary responsibility for the final content. All authors contributed to the article and approved the submitted version.

## Funding

This study was supported partly by funding of the Xinjiang Kaimeijia Biotechnology Co., Ltd., (No. 009h2702) and by the Special Research Funding for Discipline Construction in Sichuan Agricultural University (No. 03570126).

## Conflict of interest

The authors declare that the research was conducted in the absence of any commercial or financial relationships that could be construed as a potential conflict of interest.

## Publisher's note

All claims expressed in this article are solely those of the authors and do not necessarily represent those of their affiliated organizations, or those of the publisher, the editors and the reviewers. Any product that may be evaluated in this article, or claim that may be made by its manufacturer, is not guaranteed or endorsed by the publisher.
