# Supplementary material for: Dietary licorice flavonoids powder improves serum antioxidant capacity and immune organ inflammatory responses in weaned piglets
Source: Front Vet Sci. 2022 Jul 26;9:942253. doi: 10.3389/fvets.2022.942253 (PMC9360566; doi:10.3389/fvets.2022.942253)
Supplement: Supplementary file 1 [file Data_Sheet_1.docx]

***Supplementary Material***

**Supplementary Table S1 Composition of basal diet (as fed basis)^a^**

| Ingredients | Phase | |
| --- | --- | --- |
|  | 0-14d | 15-35d |
| Corn | 37.35 | 47.79 |
| Extruded corn | 18.00 | 15.00 |
| Soybean meal | 13.00 | 18.50 |
| Extruded soybean | 10.00 | 6.00 |
| Fish meal | 4.00 | 3.00 |
| Spray-dried plasma protein | 3.00 | 0.00 |
| Whey powder | 10.00 | 5.00 |
| Soy oil | 1.03 | 1.08 |
| CaHPO_3_ | 0.78 | 0.66 |
| Limestone | 0.95 | 0.90 |
| NaCl | 0.30 | 0.30 |
| L-lysine HCl | 0.32 | 0.39 |
| DL-Methionine | 0.16 | 0.20 |
| L-Threonine | 0.11 | 0.16 |
| L-Tryptophan | 0.00 | 0.02 |
| Vitamin/trace element Premix ^1^ | 1.00 | — |
| Vitamin/trace element Premix ^2^ | — | 1.00 |
| Total | 100 | 100 |
| Nutrient composition (%) | |  |
| DE Kcal/kg | 3542.00 | 3490.00 |
| CP | 20.56 | 18.88 |
| Ca | 0.80 | 0.70 |
| STTD-digestible P | 0.40 | 0.34 |
| SID-Lys | 1.35 | 1.24 |
| SID-Met | 0.39 | 0.36 |
| SID-Thr | 0.79 | 0.73 |
| SID-Trp | 0.23 | 0.20 |

^1^The premix consist of mineral and vitamin. Provided (per kg of diet): Zn (ZnSO_4_·H_2_O), 100 mg; Mn (MnSO_4_·H_2_O), 4 mg; Fe (FeSO_4_·7H_2_O), 100 mg; Cu (CuSO_4_·5H_2_O), 6 mg; I (KI), 0.14 mg; Sodium selenite, 0.3 mg; Choline chloride, 500 mg; Vitamin A, 16450 IU; Vitamin D_3_, 4700 IU; Vitamin E, 35.25 IU; Vitamin K_3_, 4.7 mg; Vitamin B_1,_ 4.7 mg; Vitamin B_2_, 11.75 mg; Vitamin B_6_, 7.05 mg; Vitamin B_12_, 0.047 mg; Nicotinamide, 47 mg; D-pantothenic acid, 23.5 mg; Folic acid, 2.35 mg; D-biotin, 0.19 mg.

^2^The premix consist of mineral and vitamin. Provided (per kg of diet): Zn (ZnSO_4_·H_2_O), 80 mg; Mn (MnSO_4_·H_2_O), 3 mg; Fe (FeSO_4_·7H_2_O), 100 mg; Cu (CuSO_4_·5H_2_O), 5 mg; I (KI), 0.14 mg; Sodium selenite, 0.25 mg; Choline chloride, 400 mg; Vitamin A, 10500 IU; Vitamin D_3_, 3000 IU; Vitamin E, 22.5 IU; Vitamin K_3_, 3 mg; Vitamin B_1_, 3 mg; Vitamin B_2_, 7.5 mg; Vitamin B_6_, 4.5 mg; Vitamin B_12_, 0.03 mg; Nicotinamide, 30 mg; D-pantothenic acid, 15 mg; Folic acid, 1.5 mg; D-biotin, 0.12 mg.

^a^ The BD was formulated to meet the nutrient requirements of piglets according to the NRC (2012), and was reported previously in our laboratory (Chen *et al.*,2018), <https://jasbsci.biomedcentral.com/articles/10.1186/s40104-018-0275-8/tables/1>, this article is an open access article distributed under the terms of the [Creative Commons CC BY](https://creativecommons.org/licenses/) license, which permits unrestricted use, distribution, and reproduction in any medium, provided the original work is properly cited. <https://s100.copyright.com/AppDispatchServlet?title=Effects%20of%20dietary%20Clostridium%20butyricum%20supplementation%20on%20growth%20performance%2C%20intestinal%20development%2C%20and%20immune%20response%20of%20weaned%20piglets%20challenged%20with%20lipopolysaccharide&author=Ling%20Chen%20et%20al&contentID=10.1186%2Fs40104-018-0275-8&copyright=The%20Author%28s%29.&publication=2049-1891&publicationDate=2018-08-23&publisherName=SpringerNature&orderBeanReset=true&oa=CC%20BY%20%2B%20CC0>.

**Supplementary Table S2 Primers used for the RT-qPCR of the target and reference genes**

| Gene | Accession number | Primer pairs (5′ to 3′ direction) | Product  size (bp) |  |
| --- | --- | --- | --- | --- |
| Housekeeping control genes | | | | |
| *β-ACTIN* | AY550069 | F: CCCAAAGCCAACCGTGAGAA  R: CCACGTACATGGCTGGGGTG | 70 |  |
| *GAPDH* | NM_001206359.1 | F: CGTCCCTGAGACACGATGGT  R: CCCGATGCGGCCAAAT | 74 |  |
| Inflammation-related genes | | | | |
| *ICAM-1* | NM_213816.1 | F: GGAGGTGCTGAAATCTCAATGTG  R: ACCTTCATGGAGCCTCCTTTG | 65 |  |
| *IL-1β* | NM_214055.1 | F: TCTGCCCTGTACCCCAACTG  R: CCAGGAAGACGGGCTTTTG | 64 |  |
| *IL-2* | NM_213861.1 | F: GCCATTGCTGCTGGATTTACA  R: TGGAGAGATCAGCATTCTCGTAATT | 68 |  |
| *IL-6* | NM_001252429.1 | F: ATGCTTCCAATCTGGGTTCAA  R: CACAAGACCGGTGGTGATTCT | 61 |  |
| *IL-8* | NM_213867.1 | F: GCAAGAGTAAGTGCAGAACTTCGA  R: GGGTGGAAAGGTGTGGAATG | 62 |  |
| *IL-10* | NM_214041.1 | F: CAGATGGGCGACTTGTTGCT  R: GGCAACCCAGGTAACCCTTAA | 64 |  |
| *INOS* | NM_001143690.1 | F: AGAGCCAGAAGCGCTATCATG  R: CCCACTGCCCCCTCCTT | 76 |  |
| *MCP*-1 | NM_214214.1 | F: GCAAGTGTCCTAAAGAAGCAGTGA  R: GCTTGGGTTCTGCACAGATCT | 69 |  |
| *TNF-α* | NM_214022.1 | F: CGACTCAGTGCCGAGATCAA  R: CCTGCCCAGATTCAGCAAAG | 58 |  |

GAPDH: glyceraldehyde-3-phosphate dehydrogenase; ICAM-1: intercellular adhesion molecule 1; IL-1β: interleukin 1β; IL-2: interleukin 2; IL-6: interleukin 6;IL-8: interleukin 8; IL-10: interleukin 10; INOS: inducible nitric oxide synthase; MCP-1: monocyte chemoattractant protein 1; TNF-α: tumour necrosis factor α.
